# Probiotic *Weissella cibaria* LAB_Weis_Camel_L4 mitigates *Escherichia coli*-induced enteritis *via* competitive exclusion and microbiota modulation

**DOI:** 10.3389/fimmu.2025.1642209

**Published:** 2025-11-03

**Authors:** Wanjing Jin, Mengfei Zhang, Xueqin Lan, Ying Huang, Yixin Bai, Yingchao Li, Chenyang Shi, Yaolong Song, Lei Wang, Yi Zhang, Wei Zhang, Gulina Aishan, Mingyang Geng, Zhanqiang Su, Jinxin Xie, Panpan Tong

**Affiliations:** ^1^ College of Veterinary Medicine, Xinjiang Agricultural University, Urumqi, China; ^2^ Xinjiang Key Laboratory of New Drug Research and Development for Herbivores, Urumqi, China; ^3^ Key Lab Animal Bacteriology, Ministry of Agriculture, College of Veterinary Medicine, Nanjing Agricultural University, Nanjing, China; ^4^ lli Kazakh Autonomous Prefecture General Animal Husbandry Station, Xinjang Uighur Autonomous Region, Yining, China

**Keywords:** *Weissella*, probiotic, *E. coli*, gut microbiota, antibiotic alternative

## Abstract

**Background:**

Pathogenic *Escherichia coli* (*E. coli*), a significant zoonotic pathogen, contributes to considerable economic losses worldwide by causing enteric disease in neonatal animals. The therapeutic efficacy of conventional antibiotics is increasingly undermined by the development of antimicrobial resistance and perturbations in ecological homeostasis. This study introduces a novel probiotic-based intervention, systematically assessing the therapeutic potential of the newly isolated *Weissella* strain LAB_Weis_Camel_L4 in a mouse model of *E. coli*-induced enteritis. Furthermore, it investigates the underlying mechanism through which this probiotic modulates intestinal homeostasis, focusing on the “microbiota–gut–immunity” pathway.

**Methods:**

In this study, the *Weissella* strain LAB_Weis_Camel_L4 was systematically isolated and identified, followed by a comprehensive *in vitro* evaluation of its probiotic properties, including growth kinetics, acid production, and tolerance to acidic pH and bile salts. Genomic analyses were performed to assess safety at the molecular level. An enteritis mouse model induced by pathogenic *E. coli* was then established to evaluate the *in vivo* safety and therapeutic efficacy of LAB_Weis_Camel_L4 through histopathological examination. Furthermore, 16S rRNA sequencing was performed to characterize alterations in gut microbiota composition following probiotic intervention.

**Results:**

A novel *Weissella* strain, LAB_Weis_Camel_L4, was identified and showed strong probiotic characteristics. *In vitro* assays revealed high gastrointestinal tolerance (survival rate > 80%) and significant antibacterial activity (inhibition zones ranging from 12.57 to 16.76 mm). Genomic analysis verified its safety, with no detectable antibiotic resistance or virulence-associated genes. *In vivo* studies demonstrated that LAB_Weis_Camel_L4 significantly decreased mortality in *E. coli*-infected mice (*p* < 0.01), mitigated intestinal inflammation, and suppressed pathogenic colonization by modulating gut microbiota composition, highlighting its therapeutic potential.

**Conclusions:**

*Weissella* LAB_Weis_Camel_L4 significantly attenuates *E. coli*-induced intestinal inflammation and promotes mucosal barrier restoration *via* dual mechanisms involving microbiota modulation and competitive exclusion. Its potent microecological antagonistic activity and capacity to maintain intestinal homeostasis position it as a strong probiotic candidate for antibiotic substitution.

## Introduction

*Escherichia coli* (*E. coli*), a Gram-negative opportunistic pathogen, is a common resident of the intestinal microbiota in both humans and animals (1). However, certain pathogenic strains, such as O157:H7 and O104:H4, express virulence factors that provoke inflammatory responses in the gastrointestinal tract, leading to symptoms including diarrhea, vomiting, and fever. These strains represent major contributors to infectious diarrhea on a global scale (1, 2). Recent epidemiological findings indicate that the evolving pathogenicity and cross-species transmissibility of *E. coli* constitute a growing public health concern, associated with increased morbidity and mortality (1, 3). In the context of livestock, *E. coli* infections, particularly those causing enteritis in ruminants, result in persistent diarrhea and significantly compromise the growth and survival of neonatal animals (4). Economic analyses estimate that neonatal diarrhea alone inflicts billions of dollars in annual losses on the global livestock industry (4). Although antibiotics are widely employed for treatment, their prolonged use has introduced significant challenges (4, 5). These include the accumulation of drug residues in animal-derived food products (6), disruption of intestinal microbial homeostasis (7), and, most critically, the emergence and dissemination of multidrug-resistant (MDR) *E. coli*, which has undermined the effectiveness of conventional antimicrobial therapies (8). The World Health Organization (WHO) has designated drug-resistant *E. coli* as a “critical priority pathogen,” emphasizing the urgent need for alternative strategies (9).

Advancements in microbiome research have highlighted the therapeutic potential of microecological agents that target the host-microbiota interface. By restoring microbial equilibrium, these interventions offer a promising avenue to address the limitations of current antibiotic-based approaches and improve the prevention and management of infectious enteritis in animal populations.

In light of the escalating global crisis of antibiotic resistance, significant obstacles persist in the diagnosis, treatment, and eradication of drug-resistant bacterial infections, highlighting the urgent need for alternative therapeutic strategies (9). Probiotic therapy has emerged as a promising approach due to its distinct capacity to regulate the microecological environment. The development of probiotics as therapeutic agents is thus critical for mitigating the threat posed by drug-resistant pathogens to both humans and animals (10).

Evidence indicates that specific strains of *lactic acid bacteria* (LAB) exert probiotic effects through multiple mechanisms, including competitive inhibition of pathogenic colonization—such as antimicrobial peptide secretion and niche competition (11); enhancement of intestinal barrier integrity by upregulating tight junction protein expression (12); restoration of gut microbiota composition (13); and immunomodulatory actions, including regulation of T-cell responses and modulation of pro- and anti-inflammatory cytokine balance (14–16). These attributes underscore the potential of LAB as a valuable adjunct in the treatment of intestinal disorders.

The probiotic properties of LAB are strongly influenced by their evolutionary ecological niches. Camels, which are adapted to extreme environments, possess gut microbiota with distinctive stress-resilient traits, including tolerance to bile salts, resistance to extreme pH, and specialized gene clusters encoding enzymes such as β-glucosidases that aid in the degradation of plant fibers. These adaptations also support the biosynthesis of host-specific metabolites, conferring camel-derived probiotics with unique advantages for improving ruminant health.

Traditionally, probiotic formulations have been dominated by species from the genera Lactobacillus and Bifidobacterium (17). However, recent research has highlighted *Weissella* as a promising probiotic candidate, noted for its capacity to produce exopolysaccharides and modulate the Th1/Th2 immune balance, suggesting potential applications in both the food and pharmaceutical sectors (18). These advancements offer new perspectives for the development of precision probiotic therapies tailored to specific hosts and pathological conditions.

This study aimed to isolate and characterize a potential probiotic Weissella strain, LAB_Weis_Camel_L4, from the camel gastrointestinal microbiota, and to evaluate its safety, host adaptability, and therapeutic efficacy in a mouse model of pathogenic *E. coli* infection.

## Materials and methods

### Isolation and characterization of camel-derived *Weissella*

**Figure 1:**
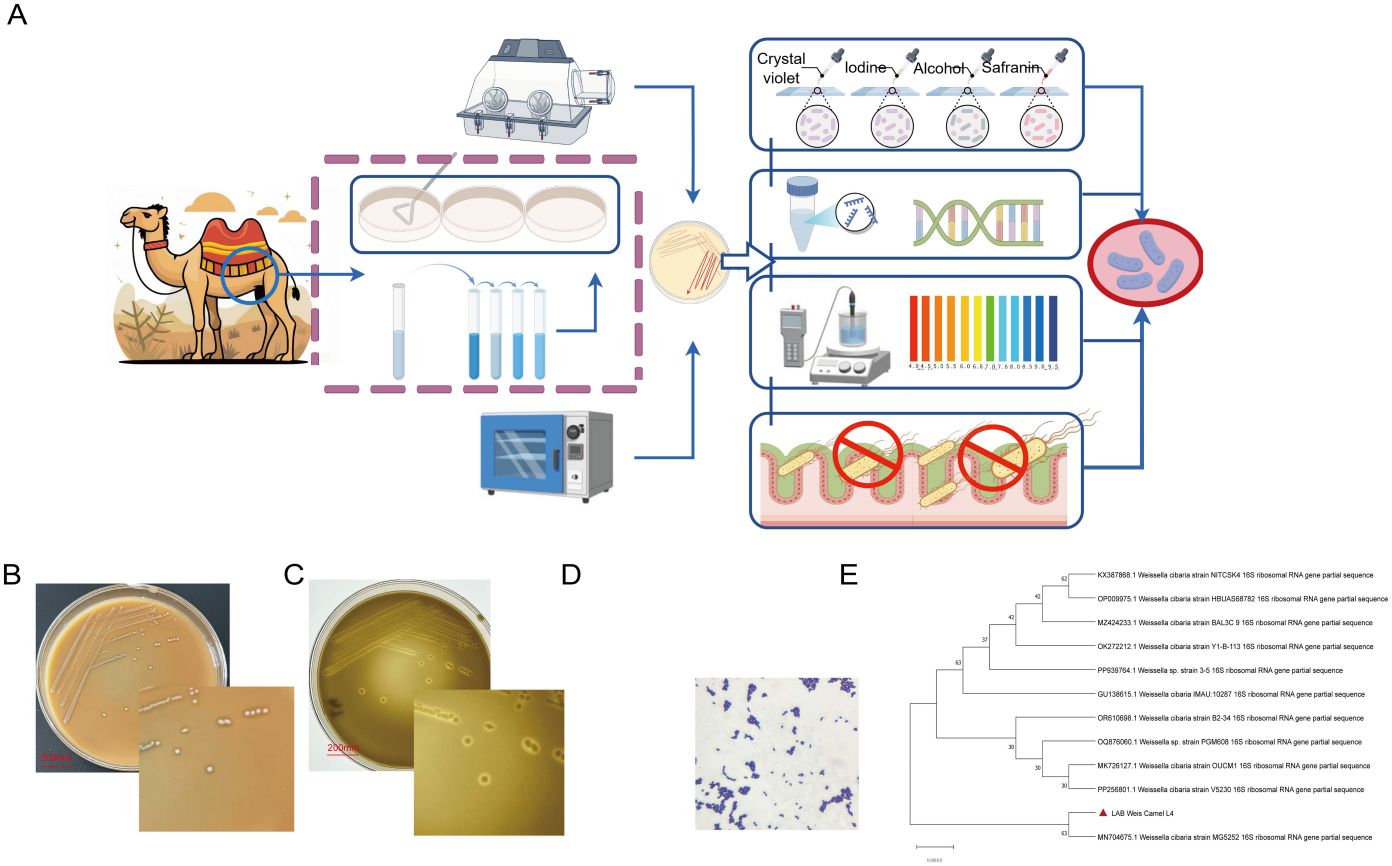
*Weissella cibaria* LAB_Weis_Camel_L4 isolation and identification. **(A)** Lactic acid bacteria dissociation identification process schematic diagram. **(B)** Formation of milky-white, smooth, circular colonies on MRS agar. **(C)** Calcium-dissolving zone (halo formation). **(D)** Gram-positive short bacilli. **(E)** Phylogenetic tree constructed based on the 16S rRNA gene.

Fresh rumen content samples from camels were subjected to 10-fold serial dilutions, and 100 μL aliquots were plated onto MRS agar supplemented with CaCO_3_. Plates were incubated anaerobically at 37 °C for 24 hours (h) (**Figure 1A**). Colonies showing clear calcium-dissolving halos were selected and enriched in MRS broth, followed by streak purification to obtain pure isolates. Preliminary identification involved Gram staining (indicating Gram-positive morphology) and physiological-biochemical characterization, including motility, oxygen tolerance, and carbohydrate fermentation/metabolic profiling (19). Definitive identification was performed through 16S rRNA gene sequencing.

### Growth and acid production curves

An overnight culture of *Weissella* was inoculated into MRS broth at 0.1% (v/v) and incubated anaerobically at 37 °C. Samples were collected at 2-h intervals to measure optical density (OD) at 600 nm (OD_600_) for growth assessment and pH for monitoring acid production. Growth kinetics and acidification profiles were then plotted to evaluate the strain’s metabolic activity over time.

### Acid and bile salt tolerance growth curve

*Weissella* strains were initially cultured on MRS agar supplemented with CaCO_3_ and incubated at 37 °C for 18 h. Individual colonies were then transferred into 5 mL of MRS broth and incubated for another 24 h. The resulting cultures were used to inoculate MRS media adjusted to pH values of 2, 3, 4, 5, and 6 (control), and supplemented with bile salt concentrations of 0% (control), 0.05%, 0.1%, 0.15%, and 0.2%.

Each treatment consisted of 5 mL of the modified medium inoculated with 1% (v/v) of the bacterial suspension, followed by incubation at 37 °C. At designated time points (0, 2, 4, 6, 8, 10, 12, and 14 h), 200 μL samples were collected in triplicate into a 96-well plate. OD at 600 nm (OD_600_) was measured using a microplate reader to generate time-absorbance growth curves, therefore assessing the strain’s growth dynamics under varying pH and bile salt conditions.

### Determination of H_2_O_2_ production

Autoclaved MRS agar, cooled to approximately 50 °C, was supplemented with 333 µL of 3,3′,5,5′-tetramethylbenzidine (TMB, 15 mg/mL) and 200 µL of horseradish peroxidase (HRP, 10 mg/mL). The components were thoroughly mixed before solidification. Once solidified, *Weissella* strains were streaked onto the prepared plates and incubated anaerobically at 37 °C for 48–72 h. Hydrogen peroxide production was assessed based on the chromogenic reaction between TMB and HRP (20).

### Hemolytic activity assay

Freshly cultured bacterial strains were uniformly inoculated onto BHI agar plates supplemented with 5% defibrinated sheep blood and incubated at 37 °C for 24 h. Hemolytic activity was evaluated by visually inspecting the colonies for hemolytic zones. Hemolysis was categorized as α-hemolysis (partial hemolysis with greenish discoloration), β-hemolysis (complete hemolysis with clear zones), or γ-hemolysis (absence of hemolysis) (21). All assays were performed in triplicate to ensure reproducibility.

### Drug sensitivity assay

The antimicrobial susceptibility of *Weissella* strains was assessed using the disk diffusion method against a panel of 25 antibiotics spanning eight classes. The tested agents included β-lactams (ampicillin, amoxicillin, cefotaxime, piperacillin, ceftazidime, cefepime, ampicillin-sulbactam, piperacillin-tazobactam, aztreonam, cefoxitin, ceftriaxone), aminoglycosides (gentamicin, amikacin, streptomycin, neomycin), quinolones (ciprofloxacin, levofloxacin), tetracyclines (tetracycline), sulfonamides (trimethoprim-sulfamethoxazole), phenicols (florfenicol, chloramphenicol), macrolides (azithromycin), and phosphonic acids (fosfomycin).

For antimicrobial susceptibility testing, overnight cultures were centrifuged and washed twice with phosphate-buffered saline (PBS), then adjusted to a concentration of 1 × 10^7^ CFU/mL. A 100 μL suspension was evenly spread onto MRS agar plates. Sterile antibiotic disks were applied to the surface using forceps, and plates were incubated at 37 °C for 24 h. Inhibition zone diameters were measured with a Vernier caliper for precision. *E. coli* ATCC 25922 was used as the quality control strain. Susceptibility classifications—sensitive, intermediate, or resistant—were determined based on established interpretive criteria (12, 13, 22).

### Next-generation sequencing

Bioinformatics approaches were applied to comprehensively analyze the *Weissella* genome. Initially, raw sequencing reads were assembled using SPAdes. Gene annotation and functional predictions were later carried out using ABRicate and the Center for Genomic Epidemiology (CGE) platform. Species identification was performed *via* rapid K-mer analysis using the KmerFinder tool (https://cge.food.dtu.dk/services/KmerFinder/). Prediction of virulence factors was achieved through the VirulenceFinder database (https://cge.food.dtu.dk/services/VirulenceFinder/), while antibiotic resistance genes were identified by screening the Comprehensive Antibiotic Resistance Database (CARD) (https://card.mcmaster.ca/). All analyses employed default parameters to maintain result consistency and reproducibility.

### *In vitro* antibacterial assay

The antibacterial activity of *Weissella* was assessed using the agar diffusion method. Test strains included standard bacteria: *E. coli* ATCC 25922, *Staphylococcus aureus* ATCC 29213, *Salmonella* ATCC 13076, *Salmonella Typhimurium* ATCC 14028, as well as three *enterohemorrhagic E. coli* (EHEC) strains maintained in our laboratory. All bacterial suspensions were adjusted to 0.5 McFarland standard turbidity after 24 h of incubation at 37°C. A 100 μL aliquot of each suspension was evenly spread onto Mueller-Hinton (MH) agar plates. Wells were created and sealed with 20 μL of MH medium. The experimental wells received 200 μL of *Weissella* culture supernatant, whereas control wells were treated with an equal volume of MRS broth. Each condition was tested in triplicate. During the 24-h incubation at 37 °C, 100 μL of bacterial suspension was replenished every 2 h. Inhibition zone diameters were measured with a Vernier caliper at the end of incubation. Antibacterial activity was categorized as follows: less than 8 mm, negative (-); 8–12 mm, weak inhibition (+); 12–16 mm, strong inhibition (++); greater than 16 mm, very strong inhibition (+++) (23).

### Evaluation of the safety of *Weissella* in mice and its preventive efficacy against *E. coli* infection

In this study, six-week-old male Kunming mice were maintained under specific pathogen-free conditions. All procedures were approved by the Animal Ethics Committee of Xinjiang Agricultural University and conducted according to the ARRIVE guidelines (No. 2023050). Following a one-week acclimation, mice were randomly assigned into four groups (n = 15 per group): Group A (Safety Evaluation, L4) received *Weissella* L4 (200 μL, 1 × 10^8^ CFU/day) *via* oral gavage for 13 consecutive days; Group B (Prevention, L4 + *E. coli*) received the same treatment as Group A and were then challenged with *E. coli* (200 μL, 1 × 10^8^ CFU/mL) on day 13; Group C (Challenge, *E. coli*) was challenged only with *enterohemorrhagic E. coli* on day 13; Group D (Control) was administered an equal volume of sterile saline daily. Body weight and clinical signs were monitored daily. Mice that died during the study or were euthanized on day 15 underwent immediate aseptic collection of heart, liver, spleen, lungs, kidneys, reproductive organs (uterus/testes), brain, and intestinal tissues (duodenum, jejunum, cecum, colon). Following the removal of adipose tissue, organ weights were recorded to calculate organ-to-body weight ratios. All tissues were fixed in 4% paraformaldehyde, embedded in paraffin, sectioned, and stained with hematoxylin and eosin for comprehensive histopathological analysis of microscopic lesions in the heart, liver, spleen, lungs, kidneys, and intestinal tract.

### Gut microbiota 16S rRNA sequencing

Gut microbiota composition changes were assessed using full-length 16S rRNA sequencing. Twelve duodenal content samples (three per group) were selected for analysis. Genomic DNA was extracted from samples stored at -80 °C and used as a template for amplifying the full-length 16S rRNA gene. Paired-end sequencing (PE 250) was conducted on the Illumina NovaSeq 6000 platform using 16S V4-specific primers (515F/806R) and V3-V4 primers for amplification. PCR products were purified with magnetic beads, quantified by enzyme-linked immunosorbent assay (ELISA), and pooled in equimolar amounts. Target fragments were confirmed by 2% agarose gel electrophoresis and recovered. Following library construction, quality control was performed using Qubit fluorometry and quantitative PCR. Libraries meeting quality standards were subjected to sequencing.

Bioinformatics analysis of 16S/ITS sequencing data began with demultiplexing raw reads using barcode sequences. FLASH (v1.2.11) was applied to assemble reads into Raw Tags. Primer sequences were removed with Cutadapt, followed by quality filtering using fastp (v0.23.1) to obtain Clean Tags (24). Chimeric sequences were identified and removed by alignment against the Silva database (16S/18S rRNA) and the UNITE database (ITS), producing Effective Tags. Denoising was performed with the DADA2 or Deblur modules within QIIME2, generating final Amplicon Sequence Variants (ASVs) and a corresponding feature table. Taxonomic annotation was conducted in QIIME2, and genus-level species accumulation curves were plotted to assess sampling adequacy. Using ASV annotation results, a taxonomic abundance table spanning kingdom to species levels was constructed, emphasizing differences in microbial community structure at the phylum, family, and species levels. This analysis revealed dynamic alterations in gut microbiota composition among treatment groups.

### Statistical analysis

Statistical analyses were performed using GraphPad Prism software. Intergroup comparisons were performed using t-tests, with significance defined as *p* < 0.05. Alpha diversity metrics included the Shannon and Simpson indices to assess microbial diversity, and the Chao index to estimate bacterial richness. Linear discriminant analysis (LDA) was applied to evaluate the effect size of species abundance differences. Significance levels were indicated by symbols: * for *p* < 0.05 and ** for *p* < 0.01.

## Results

### Isolation and identification of LAB_Weis_Camel_L4 from camel

A strain designated LAB_Weis_Camel_L4 was isolated from camel rumen content samples. After 24 h of incubation at 37 °C on MRS agar, the bacterium produced milky-white, smooth colonies (**Figure 1B**) and displayed a calcium-dissolving zone (**Figure 1C**). Microscopic examination revealed Gram-positive short rod morphology (**Figure 1D**). Biochemical assays confirmed positive fermentation of glucose, maltose, and other carbohydrates (**Table 1**). Identification based on 16S rRNA gene sequencing and phylogenetic analysis (**Figure 1E**) classified the isolate within the genus *Weissella*.

**Table 1:** The biochemical identification results of the bacterial strains.

Characteristic	Results judgement		Cultivation time (hour)	LAB_Weis_Camel_L4
	Positive	Negative		
Seven Leaf Agents	Yellow or black	Purple or purple gray	24-48	+
Fiber Disaccharides	Yellow	Purple or purple gray	24-48	+
Maltose	Yellow	Purple or purple gray	24-48	+
Glycol alcohol	Yellow	Purple or purple gray	24-48	+
Hydrazine	Yellow	Purple or purple gray	24-48	+
Pear Alcohol	Yellow	Purple or purple gray	24-48	+
Sucrose	Yellow	Purple or purple gray	24-48	+
Cotton Sugar	Yellow	Purple or purple gray	24-48	+
Stevia	Yellow	Purple or purple gray	24-48	+
Lactose	Yellow	Purple or purple gray	24-48	+

### *Weissella cibaria* LAB_Weis_Camel_L4 growth characteristics

**Figure 2:**
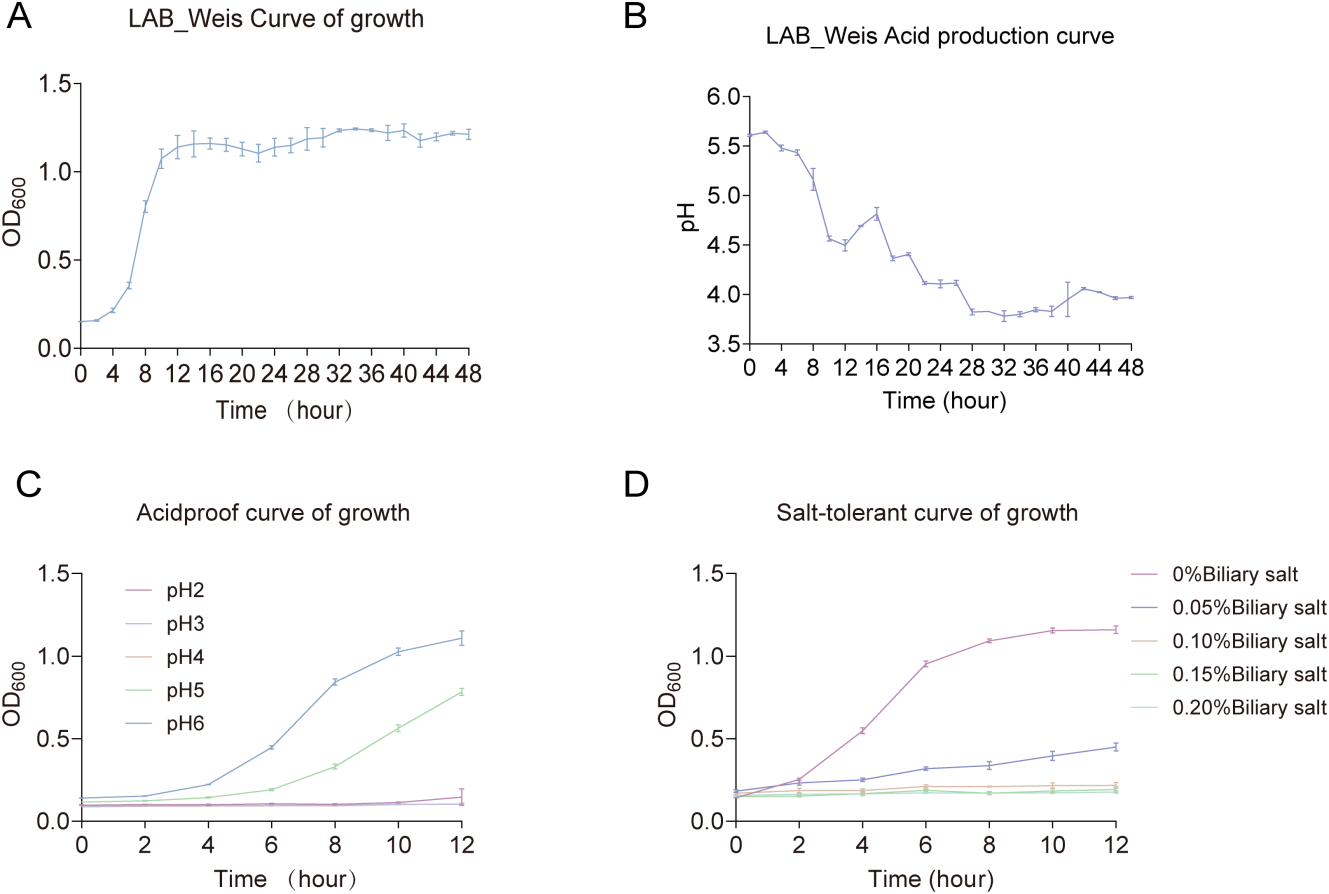
*Weissella cibaria* LAB_Weis_Camel L4 growth characteristics. **(A)** Growth curve. **(B)** Acid production curve. **(C)** Acid tolerance growth curve. **(D)** Choline tolerance growth curve.

The growth curve of LAB_Weis_Camel_L4 displayed a characteristic sigmoid pattern, consisting of a lag phase (0–2 h), a logarithmic phase (2–16 h), and entry into the stationary phase by 24 h (**Figure 2A**). Throughout cultivation, the pH steadily decreased and stabilized below 4.2 after 24 h (**Figure 2B**). The strain displayed normal growth at pH 4–5 while showing limited growth but retaining viability at pH 2-3 (**Figure 2C**). In bile salt conditions, growth was robust at concentrations of 0.05% - 0.10%, with slowed growth but sustained viability at 0.15% - 0.20%. These findings demonstrate that LAB_Weis_Camel_L4 possesses strong acid production capacity (pH < 4.0), tolerates moderate acidity (pH 4-5), and endures low bile salt concentrations (≤ 0.15%) (**Figure 2D**), indicating its potential as a probiotic capable of surviving gastric acid and bile salt exposure to colonize the intestine and exert beneficial effects.

### *Weissella cibaria* LAB_Weis_Camel_L4 safety evaluation

**Figure 3:**
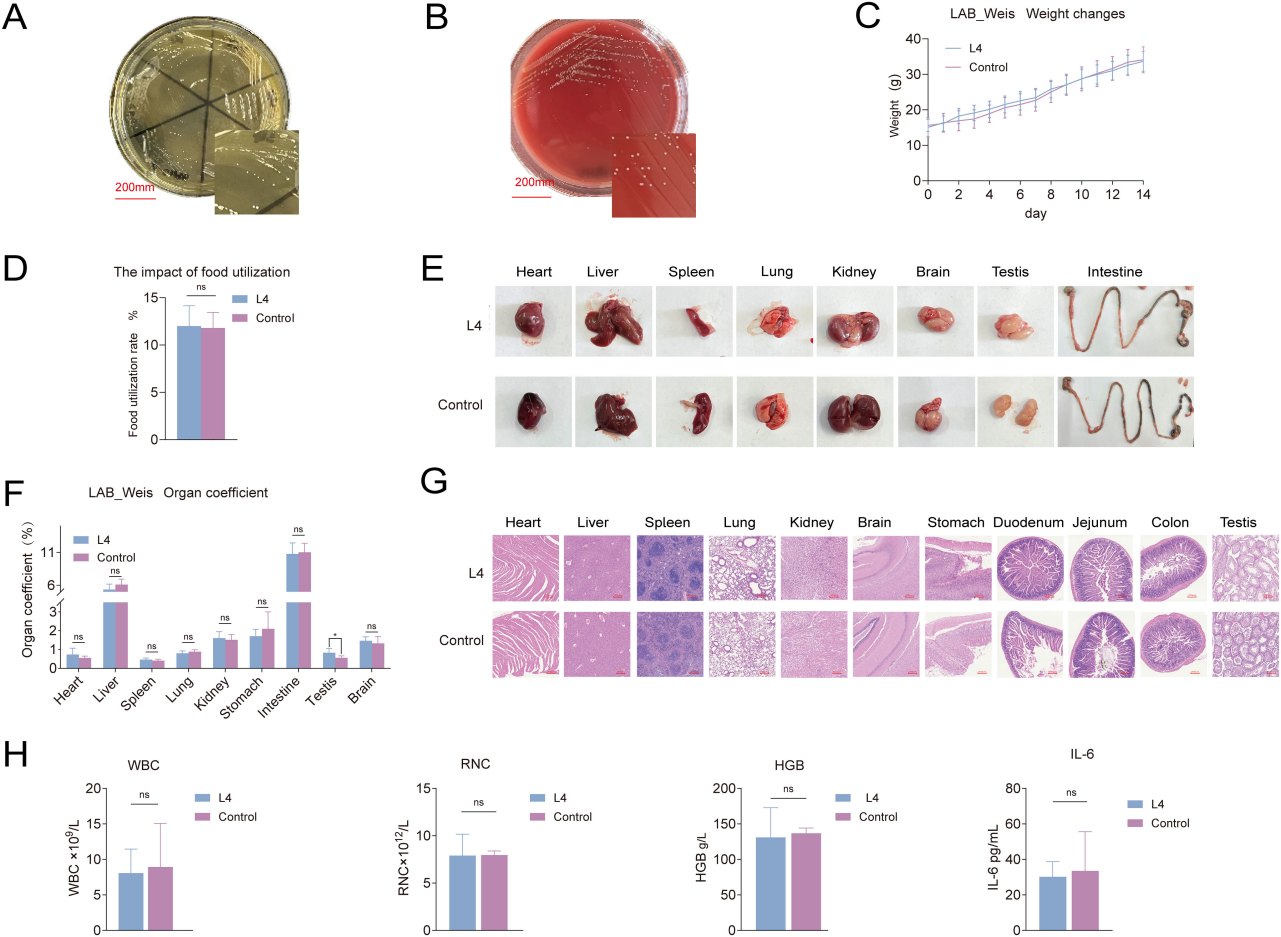
*Weissella cibaria* LAB_Weis_Camel_L4 safety evaluation. **(A)** H2O2 production assay. **(B)** No hemolysis observed. **(C)** Changes in mouse body weight. **(D)** Effect on food utilization rate in mice. **(E)** Changes in mouse tissues and organs. (F) Organto-body weight ratios in mice. **(G)** Histopathological HE changes in mouse tissues. **(H)** Hematological and inflammatory responses in mice, including effects on white blood cells WBC, hemoglobin HGB, red blood cells RBC, and the pro-inflammatory cytokine IL-6.

LAB_Weis_Camel_L4 was experimentally confirmed to be incapable of producing H_2_O_2_ *in vitro*, as indicated by milky-white colony morphology (**Figure 3A**), and displayed no hemolytic activity (**Figure 3B**). The strain demonstrated resistance to aztreonam, ciprofloxacin, streptomycin, fosfomycin, sulfamethoxazole, and cefoxitin. Genomic analysis revealed the absence of antibiotic resistance and virulence genes. It displayed potent *in vitro* antibacterial activity against standard strains, including Staphylococcus aureus, *E. coli*, Salmonella sp., as well as four EHEC isolates derived from diarrheic calves (**Table 2**).

**Table 2:** The *in vitro* antibacterial activity of LAB_Weis_Camel_L4 against standard bacterial.

Strains	Type	Antibacterial circle diameter (mm)	Decision outcomes
		8-12, +; 12-16, ++; >16, +++	
ATCC29213	*Staphylococcus aureus*	13.64	++
ATCC25922	*E. coli*	16.76	+++
ATCC14028	*Salmonella typhimurium*	13.9	++
ATCC13076	*Salmonella*	16.08	+++
DG28	EHEC	15.16	++
211	EHEC	14.31	++
G-8-1	EHEC	14.62	++
55-G-1	EHEC	13.88	++

*In vivo* assessment showed no significant difference in body weight between treated and control groups (**Figure 3C**), although food utilization efficiency was slightly increased (*p* < 0.05) (**Figure 3D**). Organ indices for the heart, liver, spleen, lungs, and kidneys remained within normal ranges, with no macroscopic lesions (**Figure 3E, F**) or histopathological alterations (**Figure 3G**). No bacterial translocation was detected in blood, spleen, liver, or kidneys, and mesenteric lymph node translocation rates did not differ significantly. Hematological parameters, including hemoglobin, white blood cell, and red blood cell counts, were maintained within normal limits, with no indications of inflammation or infection (**Figure 3H**).

### *Weissella cibaria* L4 alleviation of *E. coli*-induced enteritis in mice

**Figure 4:**
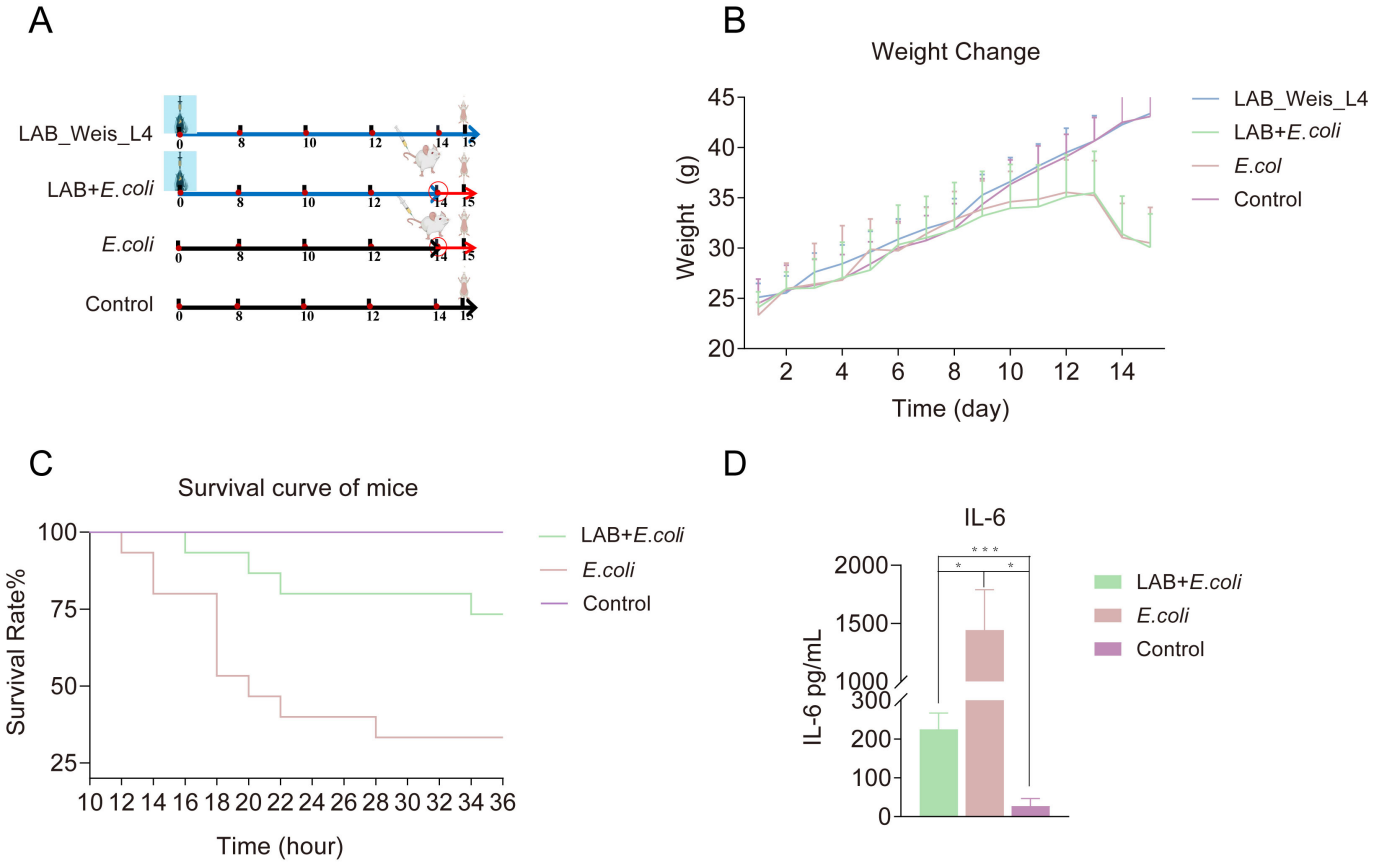
*Weissella cibaria* LAB_Weis_Camel_L4 Alleviated inflammation and prolonged the survival time of mice. **(A)** A flow chart showing the animal experiment design. **(B)** Changes in mouse body weight over 14 days. **(C)** Survival curve of mice. **(D)** Pro-inflammatory cytokine IL-6 levels in mice.

*Weissella* L4 was administered continuously to mice before the challenge with *E. coli* (**Figure 4A**). Both the L4+*E. coli* and *E. coli* groups experienced weight loss **Figure 4B**. Mortality in the *E. coli* group began at 12 hours post-challenge, whereas in the L4+*E. coli* group, mortality onset was delayed until 16 h, resulting in a 40% reduction in the final mortality rate (**Figure 4C**) significantly elevated in both groups; however, the increase was substantially lower in the L4+*E. coli* group. coli group relative to the *E. coli* group (**Figure 4D**).

**Figure 5:**
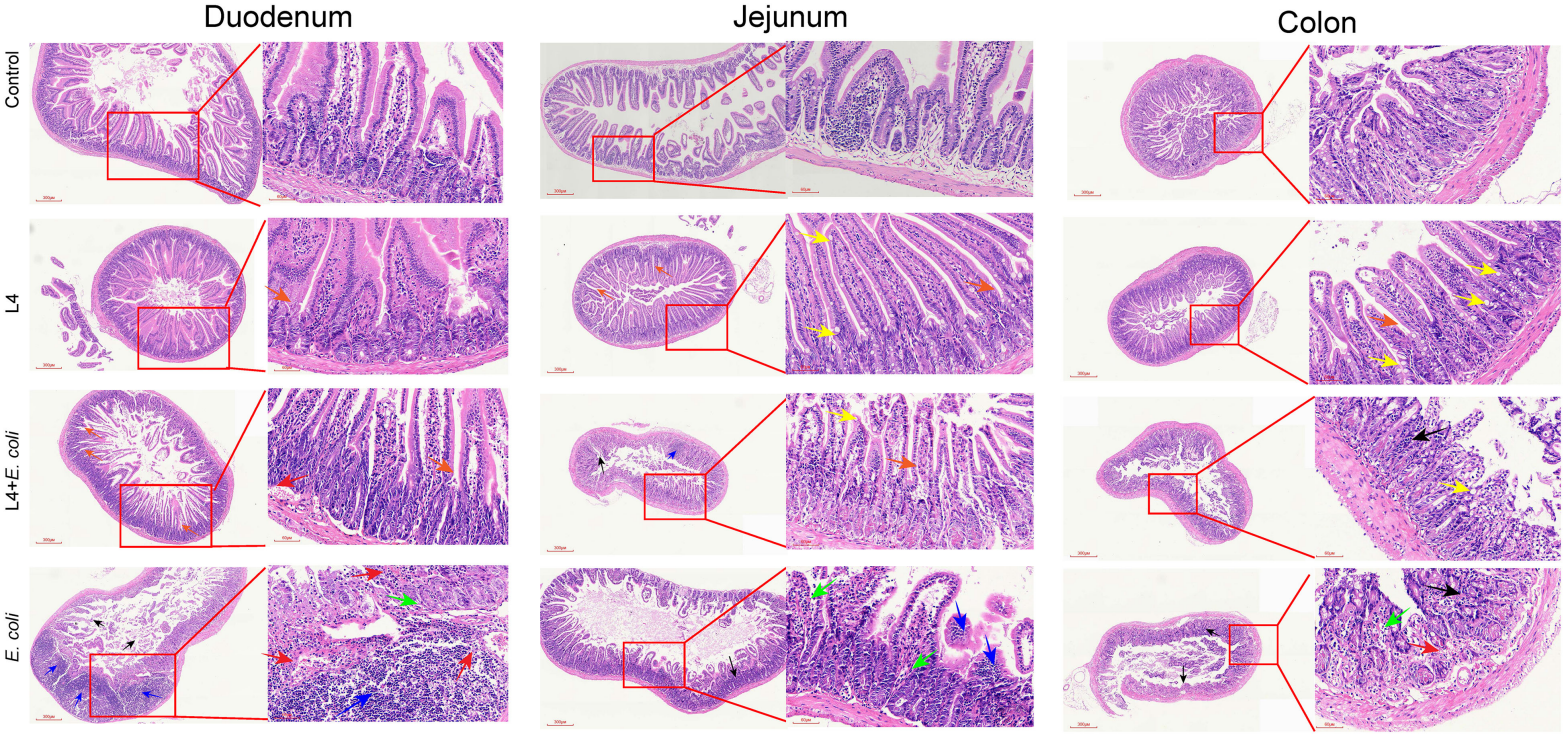
*Weissella cibaria* LAB Weis Camel L4 Alleviated intestinal pathological damage in *E. coli*-infected mice. Histopathological examination revealed: disappearance of villi and crypts (black arrows), inflammatory cell infiltration (blue arrows), loss of goblet cells (green arrows), mild focal hemorrhage (red arrows), crypts (orange arrows), and goblet cells (yellow arrows) in the duodenum, jejunum and colon of mice.

Histological analysis of duodenal tissue showed severe damage in the *E. coli* group, characterized by disorganized and fractured intestinal villi, increased crypt depth, and inflammatory cell infiltration. In comparison, the L4+*E. coli* group maintained well-organized villi, intact mucosal layers, and normal crypt architecture (**Figure 5**). In jejunal tissue, the *E. coli* group showed thinning of the intestinal wall, decreased villi density, disrupted microvilli continuity, reduced goblet cell numbers, and inflammatory infiltration, whereas the L4+*E. coli* group demonstrated improved microvilli continuity, increased goblet cell count, and restoration of the intestinal villi’s basal structure (**Figure 5**). Colon tissue in the *E. coli* group showed crypt and goblet cell loss, localized hemorrhage, and intestinal wall thickening. These alterations were significantly ameliorated in the L4+*E. coli* group, which showed restoration of crypt and goblet cell numbers, reduction of inflammatory lesions and neutrophil infiltration, and normalization of intestinal wall thickness (**Figure 5** *).*

### *Weissella cibaria* L4 modulated the gut microbiota in mice with *E. coli-*induced enteritis

**Figure 6:**
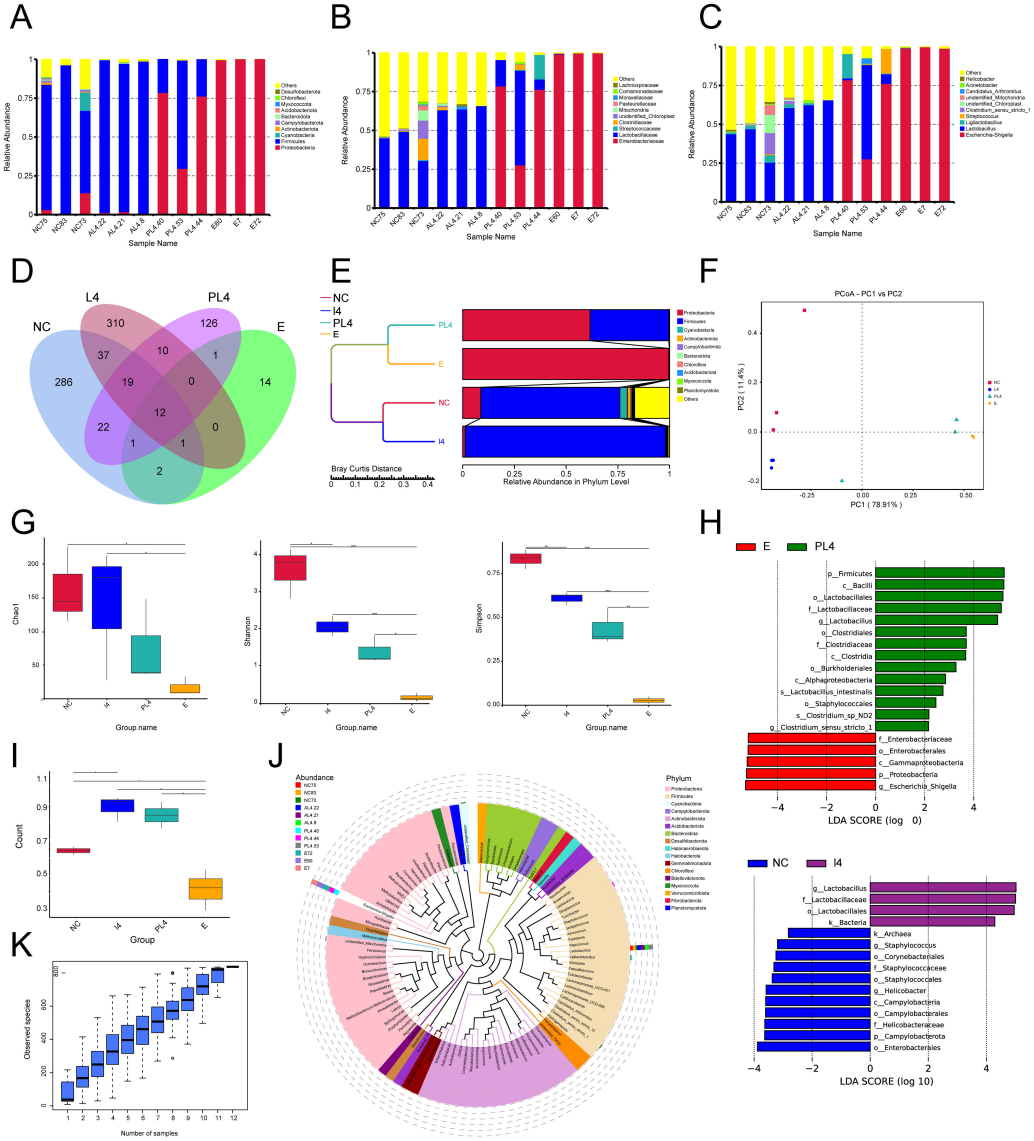
*Weissella cibaria* LAB_Weis_Camel_L4 enhance the species richness of intestinal microbiota in mice. **(A)** Species abundance at the phylum level was analyzed. **(B)** Taxonomic distribution at the family level. **(C)** Taxonomic distribution at the genus level. **(D)** Petal plot of gut microbial species richness. **(E)** UPGMA clustering tree of species abundance. **(F)** Principal Coordinates Analysis. **(G)** Alpha diversity index analysis, including Chaol, Shannon, and Simpson indices. **(H)** LEfSe analysis: LDA (Linear Discriminant Analysis) score distribution bar plot. **(I)** Beta diversity intergroup difference test. **(J)** Phylogenetic tree of species at the genus level. **(K)** Species accumulation boxplot for alpha diversity.

*Weissella cibaria* intervention significantly enhanced gut microbiota composition. At the taxonomic level, the preventive group displayed substantially greater diversity, with 60 families and 72 genera identified, compared to 12 families and 12 genera in the infected group (**Figure 6A–C**). Operational taxonomic unit (OTU) analysis revealed an increase in OTU count from 14 in the infected group to 126 in the preventive group, with the L4 group reaching 310 OTUs (**Figure 6D**). UPGMA clustering and PCoA analyses demonstrated that the intervention group maintained a healthy microbiota structure, dominated by Firmicutes, while significantly inhibiting the expansion of Proteobacteria (**Figure 6E, F**). Alpha diversity metrics (Chao1, Shannon, Simpson) indicated significant improvements in microbial richness and evenness (**Figure G**). LEfSe analysis revealed the enrichment of beneficial taxa such as *Lactobacillus* in the preventive group, whereas the infected group was dominated by pathogenic taxa, including Enterobacteriaceae (**Figure 4H**). Beta diversity analysis showed the highest microbial heterogeneity within the L4 group (*p* < 0.05) (**Figure 6I**). A genus-level phylogenetic tree confirmed that L4 treatment promoted Firmicutes colonization and suppressed Proteobacteria proliferation (**Figure 6J**). Species accumulation analysis further supported the significant increase in OTU count (*p* < 0.01) and demonstrated high data processing stability in the L4 group (**Figure 6K**).

## Discussion

Antimicrobial resistance (AMR) represents a critical global public health threat (25, 37). Recent projections estimate that AMR-related deaths could reach 10 million annually by 2050 (26). In livestock production, excessive antibiotic use has exacerbated resistance problems, adversely affecting animal health and posing potential risks to human health *via* the food chain (5, 27). This study isolated a novel *Weissella* strain (LAB_Weis_Camel_L4) from the rumen contents of Xinjiang camels, highlighting three main advantages: First, genomic analysis confirmed the complete absence of antibiotic resistance genes, therefore eliminating the risk of resistance gene transfer. Second, the strain demonstrates multiple probiotic functions, including inhibition of pathogens, modulation of immune responses, and promotion of animal growth. Third, originating from an extreme environment, this strain shows significant adaptability to gastrointestinal conditions.

As a fundamental functional component of the livestock intestinal microecosystem, LAB plays an indispensable role in maintaining intestinal homeostasis and increasing host health (28). In this study, a novel *Weissella* strain, designated LAB_Weis_Camel_L4, was successfully isolated and characterized from the intestinal microecosystem of Bactrian camels adapted to the extreme desert environment of Xinjiang. Compared to conventional strains derived from fermented foods (29, 30), this habitat-specific strain shows superior environmental adaptability and functional properties: First, it displays an exceptionally strong acid-producing capacity, lowering the culture medium pH to 3.7 ± 0.006 at the endpoint. This highly acidic environment effectively suppresses the growth of various intestinal pathogens, consistent with a key probiotic criterion whereby LAB exerts antimicrobial effects *via* organic acid metabolites (31). Second, the strain demonstrates significant tolerance to harsh gastrointestinal conditions, maintaining viability at extreme acidity (pH 2.0 - 3.0), sustaining activity for over 4 h in 0.3% bile salts, and then resuming proliferation, indicating the presence of an evolved bile acid resistance mechanism. Importantly, comprehensive *in vitro* safety evaluations confirmed the absence of hemolytic activity, fully meeting probiotic safety requirements (32). These results provide a strong theoretical and experimental basis for the future development of this strain as a novel functional feed additive.

*Weissella* inhibits *E. coli* growth and colonization through competitive exclusion. It competes with *E. coli* for critical nutrients, such as iron ions and carbon sources, thus limiting *E. coli* proliferation (33, 34). Furthermore, *Weissella* occupies adhesion sites on the intestinal mucosa, preventing *E. coli* from binding and establishing colonization (35, 36). Papud et al. demonstrated that enterotoxigenic *E. coli* (ETEC) challenge in piglets induced immunosuppression characterized by elevated pro-inflammatory cytokine IL-6, while prophylactic administration of microencapsulated probiotic strains mitigated excessive IL-6 upregulation (37). Consistently, in this study, *Weissella* intervention significantly reduced IL-6 levels (*p* < 0.001). *E. coli* infection induced IL-6 expression, which was reversed by *Weissella* supplementation, confirming its modulatory effect on IL-6 production. Although LAB_Weis_Camel_L4 effectively alleviated *E. coli*-induced enteritis by increasing microbial diversity, decreasing Proteobacteria abundance, and stabilizing gut microbiota composition, further research is required to clarify whether this probiotic directly modulates tissue inflammation. Integrative analyses combining metabolomics and host immune profiling will be critical to elucidate the underlying molecular mechanisms.

In conclusion, the probiotic *Weissella* LAB_Weis_Camel_L4 effectively modulates gut microbiota composition and mitigates intestinal inflammation in a mouse model of pathogenic *E. coli*-induced enteritis. This strain shows potential as a next-generation oral probiotic, with multifaceted functions including direct pathogen suppression, regulation of intestinal immune homeostasis, and enhancement of gut barrier repair. These results establish a theoretical foundation for developing novel microecological agents and suggest new strategies for promoting healthy animal breeding and ensuring food safety.

## Data Availability

The datasets presented in this study can be found in online repositories. The names of the repository/repositories and accession number(s) can be found below: https://www.ncbi.nlm.nih.gov/, PRJNA1254483.
